# The economic geographies of mergers and acquisitions (M&As)

**DOI:** 10.1177/0308518X231190091

**Published:** 2023-08-15

**Authors:** Liam Keenan, Dariusz Wójcik

**Affiliations:** University of Nottingham, Nottingham, UK; National University of Singapore, Singapore

**Keywords:** Mergers and acquisitions, economic geography, M&As

## Abstract

Mergers and acquisitions (M&As) are on the rise. Interlocking processes of globalization and financialization have increased their attractiveness and incentivized an upward spiral of M&A activity in recent years. This rise is profoundly spatial, as M&As reshape the geographies of production, consumption and finance, while aggravating uneven power-geometries through the concentration of corporate control. Despite this growth and inherent spatiality, economic geography research into M&As has waned. The aim this article is to demonstrate the value of M&As to economic geographers and highlight avenues for future research. This is achieved by explaining how qualitative and quantitative research into the motivations, outcomes and geographies of M&A activity can provide fresh empirical and conceptual insights surrounding wider geographical debates.

## Introduction

Mergers and acquisitions (M&As) put the global in globalization. As the leading form of foreign direct investment they play a central role in forging economic connections between distant peoples and places (Chapman, 2003). With the global economy experiencing repeated economic and ongoing environmental crises, alongside rapid and disruptive innovations in technology and artificial intelligence, M&As provide strategic and financial solutions to firms facing unprecedented and unpredictable challenges. This is because they allow firms to enter new markets, reach new consumers, create financial synergies and acquire new assets, talent and knowledge (Rompotis, 2015).

**Figure 1.**
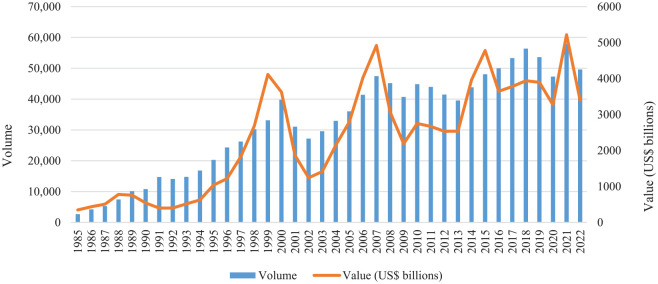
Global M&A activity, 1985–2022. Source: Institute for Mergers, Acquisitions and Alliances (2023).

As shown by Figure 1, since the 1980s there has been a gradual albeit fluctuating rise in both the value and volume of global M&A activity. Importantly, this rise is both spatially and sectorally uneven. Some sectors have experienced large increases in M&A activity (e.g. technology and finance), whereas the rise in others has been more modest (education and healthcare). Equally, some regions have been at the centre of M&A activity (e.g. North America, Europe and Asia), with others relatively bypassed by these emerging networks (e.g. South America and Africa). This uneven and exclusive geography of M&A activity has a profound impact on economic landscapes, shaping how our economies work, where decisions are made, who has access to employment opportunities and where skills, assets and wealth are concentrated.

M&As are inescapably geographical. When one firm acquires another there is always some degree of decision-making power that moves from the target to the acquirer (Zademach and Rodríguez-Pose, 2009). As the saying goes, with great power comes great responsibility, towards shareholders and other stakeholders. But acquirer firms are not beholden to act responsibly. While some firms support their newly acquired target with more staff and resources, others decide to induce cost-cutting measures and drastically rationalize operations. Regardless of how this newfound power is wielded, the process almost inevitably reconfigures the economic landscape and accelerates uneven development. In this sense, M&As are simultaneously a force of creation and destruction. Analysing their outcomes has vast potential, helping us to understand the evolving geographies of production, consumption, finance, employment and more.

In cognate fields to economic geography, including economics, international business, and strategic management, focus on M&As is broad and diverse, with research analysing the effects of M&As on firm performance (Siegel and Simons, 2010), the determinants shaping deals (Erel et al., 2012) and the relative success of M&As in terms of wealth creation (Datta et al., 1992). Despite the importance of M&As and their immutable spatiality, we believe that they have been under researched by economic geographers in recent years. There is a rich history of economic geography analysis of M&As, with studies generating novel spatial perspectives on their uneven distribution (Goddard and Smith, 1978; Green, 1990) and highlighting their role in shaping urban systems and hierarchies (Predd, 1977). More recently we have seen Chapman’s (2003) review and research agenda, Zademach and Rodríguez-Pose’s (2009) work on cross-border M&As in Europe, Böckerman and Lehto’s (2006) work on domestic deals and analysis covering M&As in the context of food (Clapp and Isakson, 2018), pharmaceuticals (Fernandez and Klinge, 2020) and finance (Contel and Wójcik, 2019), to name a few. However, and almost paradoxically, we believe that interest in M&As has waned as their proliferation across the global economy has only become more apparent.

While not suggesting that M&As have been overlooked entirely, we call for a revival in their analysis to address their poor integration into economic geography research in recent years. The aim of this *Exchange* is to demonstrate the value of M&As to economic geographers and highlight avenues for future research. We achieve this by explaining how qualitative and quantitative research into the motivations, outcomes and geographies of M&A activity can provide fresh empirical and conceptual insights in relation to wider geographical debates. The article starts by providing a methodological overview before then explaining the ways in which M&A research provides fresh insights surrounding relational geographical approaches, the interplay between agency and structure, uneven power geometries, inter-sectoral convergence and processes of financialization.

## Researching M&As

Before considering the ways in which economic geographers can benefit from M&A research, we need to understand what they are. M&As are financial transactions that involve two firms joining together and consolidating part or all of their operations. Slight variations to this definition exist and whilst there are technical differences between a merger, where firms consolidate activities in ways in which there is no defined or dominant acquirer and an acquisition, where one firm acquires and gains control over another, the terms are often used interchangeably (Coyle, 2000). M&As can be horizontal, when both firms operate in the same sector; vertical, when both firms operate in the same sector but have different roles in the production network (i.e. consider vertical integration); or inter-sectoral, when firms operate in different sectors (Green 2018; Motis, 2007). Moving from the sectoral to the spatial, M&As can also be domestic (within a country) and cross-border (international).

While M&As play an increasingly important role in the global economy, accurate information surrounding their implementation often remains obscured and typically hidden behind paywalls. In the broadest sense, M&As can be analysed from both qualitative and quantitative perspectives. Qualitative research involves listening to the voices of different types of actors involved in the implementation and impacted by the outcomes of M&A activity. From this perspective, research focuses on understanding the motivations and outcomes of M&A deals. This not only involves speaking with actors from acquirer and target firms, but also the financial and legal intermediaries that facilitate deals, the regulators that approve or block them, the unions that represent employees impacted by them, the media that frames the narrative of each deal and more. Depending on the specific research questions and geographical focus, M&As thus become a valuable *site of analysis* to shed light on wider economic processes and specific economic sectors. By adopting this approach we do not seek to abstract and decontextualize M&As, but rather understand how their motivations, implementation and outcomes intertwine with wider economic developments and structures. The value in approaching M&As as a site of analysis lies in their ability to help us explain evolving economic geographies.

Quantitative research centres on analysing M&A data in more comparative and holistic ways. This perspective focuses on analysing the geographically uneven value and volume of M&A deals over time and space. Important here are online proprietary databases, for example Bureau van Dijk’s Orbis, which provide detailed information on thousands of M&A deals but are often expensive and do not regularly include geographical data. Consequently, geocoding (e.g. ascertaining the headquarter address of all acquirer and target firms) becomes central to any sort of spatial analysis and while this can be automated, the skills and time required to do so make this a sizeable challenge. If this challenge is overcome then M&A data become a valuable *methodological tool* to explore economic geographies in diverse ways.

Different quantitative and qualitative approaches show us that M&As can simultaneously be both the *site of analysis* and the *methodological tool* to explore wider geographical processes. This hybrid dynamic also enables multi-scalar analysis, with empirical insights moving from individual decision-making and firm-level dynamics to wider sectoral and geographical transformations. While boardroom conversations might remain confidential and reliable M&A data may come at a premium, economic geographers should not be deterred. These obstacles are not impossible to overcome and as we shall now explain, both qualitative and quantitative research into M&As can yield fascinating insights.

## Relational geographies and the cultivation of networks

M&As are inherently relational. There is always one acquirer or more and one target or more. When power moves between these firms, research can conceptualize and analyse the relational spaces of economic decision-making. This relational dynamic has enormous potential for the study of networks.

Network analysis has become a central feature of economic geography. Global production networks (Yeung and Coe, 2015), globalization and world cities networks (Derudder and Taylor, 2005) and the global financial network (Haberly and Wójcik, 2022) show how economic geographers are increasingly concerned with the networked relations that underpin economic activity. While these approaches vary in terms of their outlooks and inter-disciplinary foci, they all focus on understanding the relational dynamics of economic geographies through networks. M&As are central to these networks and their conceptualizations. Acquisitions, advertently or inadvertently, allow firms to maintain, extend and manage these networks by enrolling new actors and geographies into them while simultaneously excluding and bypassing others.

Researching M&As provides unparalleled insights into the structure, geographies and evolution of economic networks. M&As not only provide a novel way to map and visualize these networks but also unravel the uneven power relations which constitute them. Which firms play leading roles in shaping and cultivating networks through M&As? What role do M&As play in constituting the relative success of some networks over others? How are target firms enrolled into these networks, and the strategic coupling involved (MacKinnon, 2012)? What are the implications for the local communities in which they are embedded? By answering these questions research can establish new ways of understanding and conceptualizing the networked relations of economic geography.

Having covered inter-firm networks, M&A also create complex intra-firm networks. Most of the world’s largest transnational corporations (TNCs) have grown through M&As. This has spawned novel ownership models (e.g. conglomerates and holding companies) where lead firms control vast webs of subsidiary firms operating in different sectors, markets and geographies. Analysing M&As helps us understand these networks of corporate ownership. By borrowing the logic of ‘follow the money’ approaches in economic geography (Hughes-McLure, 2022), research can *follow the acquisition* to trace the origins of TNCs and map the formation of complex corporate networks. Tracing acquisitions backwards allows research to understand how, when and where corporate networks merge, evolve and adapt. Ultimately, M&A analysis helps reveal the relational ties that develop between firms, cities, countries and regions.

## Agency and structure

Who calls the shots in the global economy? Are actions initiated by the agency of economic actors or are they driven by the wider structures of society that inescapably and sometimes invisibly determine our every move? Exploring the motivations behind M&A activity provides a novel opportunity to engage with these questions and contribute to wider geographical debates concerned with the complex interplay between agency and structure.

Geography is not a passive victim of M&A activity. Space, place and inescapable geographical structures inevitably shape the decision-making processes behind them. Formal institutions, including uneven political economic and regulatory landscapes (especially competition law), constrain where, when and why firms engage in M&A activity. So do informal institutions, with culture and language as significant factors in the decision-making process (Kedia and Reddy, 2016). While these geographical factors are important they are not deterministic. Agency is at the heart of these decisions, as firms engage in M&A activity to access new markets, reduce competition, improve financial synergies or acquire new technology (Motis, 2007). Aside from these more strategic motivations, deals can also be driven by a general fear of missing out on the next big thing, or equally by pride and managerial hubris (Dhir and Mital, 2012). The point is that M&As have a huge impact on economic geographies but our understanding of the messy, complex and contingent motivations behind them remains unclear and underdeveloped.

Researching these motivations, particularly when approached through a cultural political economy lens (Jessop, 2010; Jones, 2008), provides a novel opportunity to understand the intersecting roles of agency and structure in the evolution of economic landscapes. By focusing on the interplay between structure and agency in the implementation of M&As, fresh insights can be developed around the institutionally embedded, socially constructed and geographically contingent nature of economic decisions (Jessop and Oosterlynck, 2008). We can begin to answer critical questions that will shed light onto the interdependent, reciprocal and co-constitutive nature of agency and structure (Jessop, 2010; Jessop and Oosterlynck, 2008). How are M&A motivations shaped and tempered by real-world geographical structures? Which geographical narratives and imaginaries are mobilized to justify, or equally reject, these deals? Qualitative research centered on these types of questions can generate more nuanced understandings of the complex interplay between agency and structure in economic geography.

## Uneven power-geometries and the concentration of corporate control

Power is at the heart of M&As. In any given M&A deal corporate control and decision-making power moves from the target firm to the acquirer (Zademach and Rodríguez-Pose, 2009). The transactional nature of M&As means that we do not only see where power is moving to but also where power is moving from. This immediately demonstrates the value of M&A analysis in terms of understanding how power moves and concentrates throughout the global economy. Importantly, the recent increase in M&A activity raises urgent questions around monopolization, rent-seeking and aggravated power inequalities.

M&As cultivate monopolies. By subduing competition they allow firms to accumulate and protect monopoly power over space and time. There is a growing trend of monopolization across all types of sectors in the global economy, as firms use M&As to concentrate power, remove competition and protect long-term profitability (Philippon, 2019). This process has significant implications for the price of goods and services, levels of long-term investment and the conditions, remuneration and nature of work. Monopolization does not only rework power dynamics between firms but also between capital and labour, with more research required to explore the uneven impacts of increased M&A activity from the perspective of labour geographies (Edwards, 1999). While monopolies are not inevitable, as legal and regulatory frameworks play an important role in maintaining a balance between competition and monopoly (Christophers, 2016), M&As have undoubtedly accelerated this trend.

These issues are demonstrated in the case of the global food sector that has experienced unprecedented levels concentration in recent decades (Clapp et al., 2021; Hendrickson et al., 2020). Partly driven by financialization and the incursion of new financial actors who see the food sector as an opportunity to generate huge returns, corporate actors have implemented wide-ranging M&A strategies to consolidate control, increase market share, improve financial performance and redistribute accumulated wealth back to investors and shareholders (Clapp and Isakson, 2018). This concentration of power, centered on the narrow and short-term pursuit of financial returns, has exacerbated the vulnerabilities of the global food system. It has increased volatility in food prices, intensified exploitation and forms of labour precarity, created new regulatory challenges related to competition law and significantly hindered the opportunities for collective action around climate change and sustainability (Clapp and Isakson, 2018; Isakson, 2014; Muehlfeld et al., 2011). This shows how the concentration of wealth and power through M&As not only aggravates inequalities but also excludes actors from meaningfully reshaping the sectors in which they are employed.

The global food sector example exposes a contradiction between the use value and exchange value of firms as commodities to be acquired and sold, while highlighting the use of M&As as a form of ‘accumulation by dispossession’ to establish monopolies (Harvey, 2015: 133). By this we mean that firms increasingly see themselves and their competitors through narrow quantitative measures of value, prioritizing strategies that allow them to acquire competitors and protect themselves from acquisition, which undermines the wider qualitative value of firms in relation to the role they play in society. If firms increasingly prioritize M&A activity to meet their strategic goals and redistribute accumulated wealth to shareholders, what implications does this have for investments in productive activities and the local communities dependent upon them? Does this mean that the burden of innovation falls on small to medium-sized firms, while larger and better financed firms focus less on producing what they need and more on acquiring what they need? M&A analysis can help answer these questions while also revealing how and why power moves, concentrates and is exercised across different sectors and geographies. This can help generate more fluid, dynamic and relational conceptualizations of power, while also generating critical insights into processes of monopolization and uneven power-geometries.

## Inter-sectoral convergence

Economies are always in a state of change. At present, technology, artificial intelligence and to a large extent finance, are profoundly changing how goods and services are produced and consumed (Keenan et al., 2022). As innovations in these areas proliferate, the economy is experiencing increased levels of inter-sectoral convergence. Defined as the ‘blurring of boundaries between industries due to converging value propositions, technologies and markets’ (Bröring et al., 2006: 488), there has been an increase in inter-sectoral activity since the early 2000s as firms are increasingly expected to venture into new sectors and markets to develop competitive and comparative advantages (Heo and Lee, 2019; Lim, 2020). M&As underpin this process of inter-sectoral convergence as they allow firms to reach into new sectors and acquire the relevant assets, talent and knowledge needed to meet these new opportunities and challenges.

M&A activity of this kind is transforming the structure, geographies and nature of economic sectors. For example, the FinTech revolution has been underpinned by the cross fertilization of the finance and technology sectors, as new innovations transform how money moves around the world (Lai and Samers, 2020). Healthcare and pharmaceuticals have also undergone technological transformations, as investments in biotechnology and patient data systems have reconfigured operations (Danzon et al., 2007). Car manufacturers no longer just produce cars but also provide a diverse range of financial services to support consumers through loans, leasing and insurance (Borghi et al., 2013; Carmo et al., 2019). Firms could invest in internal processes of innovation and recruitment to adapt to these changes but M&As present a faster, less risky and if successful, financially efficient option. M&As therefore not only facilitate these transformations but intensify them.

Crucially, inter-sectoral convergence is temporally, sectorally and geographically uneven. Economic geographers know relatively little about which sectors are converging, the spatiality of these transformations and their socio-economic implications. M&A analysis provides an opportunity to address this deficiency. Uneven processes of inter-sectoral convergence can be better understood by conceptualizing a framework for analysis based on intra (e.g. financial sector firms acquiring financial sector firms), inward (e.g. real estate firms acquiring financial sector firms) and outward (e.g. financial sector firms acquiring real estate firms) M&A deals (Keenan et al., 2022). This framework will allow analysis to capture how, where and why the boundaries between different sectors are becoming increasingly blurred. Ultimately, M&As are both a force of consolidation *and* convergence. Analysing them will surely help economic geographers conceptualize the porous boundaries of economic sectors and reveal how this porosity is redefining the global economy.

## Financialization and the role of intermediaries

M&As require a significant amount of financial and legal expertise to be implemented. This is typically provided by firms operating in the financial and business services sector (FABS), which offer a diverse range of financial, accounting, legal and consultancy services (Wójcik, 2020). FABS firms support M&A activity in everything from valuations and negotiations to settlement contracts and shareholder agreements. FABS firms also support M&A activity through the provision of debt finance. Evidenced by the growing implementation of leveraged buyouts, debt has become a prominent feature of M&A activity, with relatively affordable and accessible finance creating new opportunities for deals. Put simply, firms do not need deep pockets to acquire competitors. Rather they need access to finance.

Alongside these more tangible roles, finance is also shaping M&A activity through pervasive yet geographically uneven processes of financialization. Broadly understood as the increasing dominance of financial motives, markets and actors throughout the global economy, financialization has reconfigured the priorities of non-financial firms by encouraging them to think and act like financial actors (Epstein, 2005). This includes operating under short-term planning horizons, incurring debt for growth and prioritizing the generation of shareholder value (French et al., 2011; van Treeck, 2009). This has established financialized modes of competition which have increased the attractiveness of M&As as they provide solutions to strategic and operational challenges while simultaneously capturing financial synergies, boosting short-term earnings and increasing shareholder value. In relation to the previous section, inter-sectoral M&As were traditionally driven by a firm’s desire to reduce risk through diversification (Lim, 2020). However financialization increasingly compels firms to seek financial opportunities irrespective of sectoral boundaries and to therefore acquire unrelated businesses to generate new returns and improve financial performance (Heo and Lee, 2019).

Importantly, nation-states are not immune from processes of financialization and play an active albeit uneven role in terms of M&A activity. Represented through the significant rise of transnational state-led investment in recent years, nation-states are directly participating in M&A activity by acquiring foreign firms (Babic et al., 2020). A diverse mixture of state-owned enterprises and sovereign wealth funds are emulating the strategies of private firms and increasingly engaging in M&A activity to generate financial returns, support long-term prosperity and access strategic industries, among other motivations (Alami and Dixon, 2022; Babic et al., 2020). At the same time, the proliferation of inward foreign investment screening shows how some nation-states are simultaneously slowing down global M&A activity (Danzman and Meunier, 2023). As M&As facilitate the movement of corporate power, they are not politically neutral. M&As are intimately entwined with geopolitical relations, and nation-states increasingly screen and scrutinize deals in order to ensure that potential takeovers do not pose risks to national security or undermine their wider geostrategic economic objectives (Alami, 2023; Danzman and Meunier, 2023). Researching M&As can therefore generate important insights into the uneven financialization of nation-states.

Some studies have begun to explore this relationship between financialization and M&As. For example, Bowman (2018) and de los Reyes (2017) reveal an increase in M&A activity throughout the mining sectors due to increased pressure to provide shareholder value. Equally, the financialization of pharmaceuticals has led to an upsurge in M&As as firms attempt to capture and monopolize patented blockbuster drugs (Fernandez and Klinge, 2020). However, more research is required to understand how financialization is increasing M&A activity across different sectors and the uneven outcomes this is creating. Ultimately, M&As provide a way of exploring the intersection of finance with the rest of the economy and its impacts on society. Analysing the motivations, outcomes and geographies of M&A activity can bring financial geography into closer dialogue with other areas of economic geography.

## Conclusion

The aim of this article has been to demonstrate the value of M&As to economic geographers and highlight avenues for future research. We have explained how qualitative and quantitative research into the motivations, outcomes and geographies of M&A activity can provide fresh empirical and conceptual insights in relation to wider geographical debates. In discussing some of the existing economic geography research into M&As, alongside outlining new and exciting ways to approach them, we hope to encourage more research which treats them both as a *site of analysis* and a *methodological tool*. There is no escaping the growing centrality of M&As to the global economy, and economic geographers are uniquely positioned to identify, understand and critique this role.
